# Blood Gas Interpretation Under Hypothermic Conditions: A Comparative Study of Alpha‐Stat and pH‐Stat in Neonatal Hypoxic–Ischemic Encephalopathy

**DOI:** 10.1155/ijpe/4574683

**Published:** 2025-12-29

**Authors:** Vardhil Gandhi, Arijit Lodha, Khorshid Mohammad, Abhay Lodha, James Scott, Yacov Rabi

**Affiliations:** ^1^ McMaster University Department of Medicine, Hamilton, Ontario, Canada, mcmaster.ca; ^2^ Faculty of Medicine & Dentistry, University of Alberta, Edmonton, Alberta, Canada, ualberta.ca; ^3^ University of Calgary Department of Pediatrics Calgary, Calgary, Alberta, Canada; ^4^ Alberta Children’s Hospital Research Institute, University of Calgary, Calgary, Alberta, Canada, ucalgary.ca; ^5^ University of Calgary Cumming School of Medicine, Calgary, Alberta, Canada, ucalgary.ca

**Keywords:** blood gas analysis, hydrogen-ion concentration, hypothermia, hypoxic–ischemic encephalopathy, induced

## Abstract

**Introduction:**

Acid‐base management in neonates with hypoxic–ischemic encephalopathy (HIE) undergoing therapeutic hypothermia (TH) can use either the “alpha‐stat” or the “pH‐stat” method, which adjusts values based on temperature.

**Objective:**

This study aimed to compare the efficacy of two blood gas analysis techniques in predicting the severity of brain injury in neonates with HIE.

**Method:**

A retrospective study was conducted on neonates over 35 weeks’ gestation who underwent TH between 2010 and 2015. Diagnostic, univariate, and multivariate analyses were performed to compare outcomes between the alpha‐sat and pH‐stat groups.

**Results:**

Adjusting for sex and age, the odds ratios for being classified as hypocapnic were 4.40 (95% CI: 1.19–16.27) using the alpha‐stat method and 2.94 (95% CI 1.15–26.48) using the pH‐stat method. The classification of patients as hypocapnic, normocapnic, or hypercapnic differed significantly between the two methods (*p* < 0.0001), with 23% of patients reclassified from the alpha‐stat to the pH‐stat method.

**Conclusion:**

Both blood gas analysis methods were similarly effective in predicting brain injury extent. However, the alpha‐stat method significantly overestimated the lowest pCO_2_ values during therapeutic hypothermia.

**Summary**

• Underestimation of pH and overestimation of pCO_2_ in alpha‐stat method may lead to overly aggressive fluid, ionotropic, and ventilatory management.
• There is no evidence to suggest that the pH‐stat method is beneficial compared to alpha‐stat method for improvement of brain perfusion in neonates who receive therapeutic hypothermia.
• Both blood gas analysis methods were similarly effective in predicting brain injury extent. However, the alpha‐stat method significantly overestimated the lowest pCO_2_ values during therapeutic hypothermia.

## 1. Introduction

Currently, therapeutic hypothermia (TH) is the only known neuroprotective strategy that significantly improves neurodevelopmental outcomes after perinatal hypoxic ischemic insult [1]. Reducing core body temperature has profound effects on physiological systems, which are optimized around a narrow range of temperature and pH [2]. In neuroprotective scenarios, the intracellular pH of smooth muscles in cerebral vasculature is particularly sensitive to changes in extracellular pH rationalizing the need for strict acid–base monitoring [3]. Arterial blood gases (ABGs) are helpful to the clinician in managing acid–base balance.

Dissolved gases in blood follow the principles of Henry’s law, which states that the partial pressure of a gas is proportional to its concentration at a given temperature and pressure. When the temperature decreases, the solubility of gases in the blood increases and the equilibrium between gas phase and dissolved gas phase is reached at a lower partial pressure. Thus, at lower core body temperatures as seen during TH, the solubility of both oxygen (O_2_) and carbon dioxide (CO_2_) increases, affecting the PaO_2_ and PaCO_2_, respectively. However, temperature change has a greater effect on PaCO_2_ than on PaO_2_ [4].

The alterations of pH and pCO_2_ can be particularly problematic when interpreting blood gas results from automated analyzers. Automated blood gas analyzers directly measure whole blood pH and PaCO_2_ by detecting the potential electric charge difference between two electrodes (thermostatically regulated at 37 °C). At the outset of testing, the analyzer assumes the sample temperature to be 37 °C instead of the patients’ actual temperature which is significantly lower [5]. When blood gas analyzer warms the sample to 37 °C, using vacuum sealed chambers, CO_2_ bound to hemoglobin, which does not directly contribute to pCO_2_ in vivo, reverts back to gas phase within the plasma and contributes to the measured pCO_2_ in vitro.

The pH varies with changes in temperature, increasing by 0.016 units for every 1 °C drop in temperature. Thus, a pH of 7.4 and pCO_2_ of 40.0 mmHg at 37 °C will correspond with a pH of 7.5 and pCO_2_ of 34 mmHg at 33°C. This may have important implications for the clinical management of infants with HIE. Using temperature correction for blood gas analysis may have a beneficial effect on brain perfusion in children who received total body hypothermia during cardiopulmonary bypass surgery, although the evidence from adult literature is controversial [6].

There are currently two main accepted methods of blood gas analysis, called alpha‐stat and pH‐stat. To date, these methods have not been compared in neonates undergoing therapeutic hypothermia for perinatal hypoxia. Both blood gas analysis methods utilize automated blood gas analyzers to measure pH, pCO_2_, and pO_2_. The electrodes of the automated blood gas analyzers are thermostatically maintained at 37 °C within a close vacuum system and assumes the temperature of the blood sample obtained from the patient is also 37 °C.

The alpha‐stat method utilizes the temperature maintained by electrodes, which is 37 °C, to calculate the corresponding pH and pCO_2_ measurements. By contrast, pH‐stat method utilizes the patient’s actual core body temperature, rather than the temperature set by the electrodes in the ABG analyzer, to calculate the corresponding pH and pCO_2_ measurements.

Usually, patients have a core body temperature around 37 °C and so the differences between the alpha‐stat and pH‐stat method may not be noticeable. However, in patients undergoing TH, because the body is cooled to between 33 and 34 °C, the temperature used to calculate the pH and pCO_2_ measurement has important clinical implications. In hypothermic patients, the alpha‐stat method will overestimate the pCO_2_, potentially leading to lower true pCO_2_ value than the targeted range of 35–45 mmHg. Meanwhile, the pH‐stat method would estimate a more accurate pCO_2_ readings, which will result in pCO_2_ values that more closely match their targeted values of 35–45 mmHg.

Alpha‐stat method assumes patient’s core body temperature is 37 °C; therefore, pCO_2_ is overestimated and true pH is higher in hypothermic neonates. However, pH‐stat method for clinical management can help clinicians target pCO_2_ at a higher range than alpha‐stat, thus shifting O_2_ dissociation curve to the right and improving tissue oxygenation by increasing pCO_2_ [7]. Based on animal studies, pH‐stat method during therapeutic hypothermia in neonates seems to be a more appropriate strategy [8]. It provides more brain protection and improves the efficiency of cooling.

This main objective of our study was to analyze blood gases of infants who received TH for 72 h to investigate whether the difference seen by temperature correction (alpha‐stat vs. pH‐stat) was associated with differences in predicting the severity of brain injury. The secondary objective was to elucidate differences in patient classification for PaCO_2_ levels (hypocapnic, normocapnic, hypercapnic) based on the alpha‐stat versus pH‐stat blood gas method.

## 2. Materials and Methods

This was a retrospective cohort study comparing the ability of the alpha‐stat (temperature uncorrected) and pH‐stat (temperature corrected) blood gas analysis methods to quantify derangements in pCO_2_ and to predict severity of brain injury in neonates with HIE following 72 h of TH.

All neonates > 35 weeks of gestation who received TH at the Foothills Medical Centre level 3 NICU and Alberta Children’s Hospital level 4 NICU in Calgary, Alberta, Canada, between 2010 and 2015 were included. Neonates with major congenital anomalies, and those whose TH therapy was discontinued before 72 h were excluded from the analysis.

We collected blood gas data from the patient charts and healthcare database. The most hypocapnic (lowest pCO_2_) blood gas during the 72‐h period of cooling was recorded for further analysis. In this study, we defined hypocapnia as pCO_2_ ≤ 35 mmHg, normocapnia as pCO_2_ between 35 and 45 mmHg, and hypercapnia as pCO_2_ ≥ 45 mmHg. Rectal temperatures, documented in the nursing charts hourly during TH, were collected along with the blood gas result. Magnetic resonance imaging (MRI) scans of the brain, conducted at the completion of 72 h of cooling (between days 3 and 5), were analyzed by an experienced neuroradiologist who was blind to the infant’s clinical signs of moderate or severe encephalopathy and laboratory information. The severity of brain injury was graded as “moderate” or “severe” according to the Barkovich Criteria [9].

We used brain MRI because it serves as a short‐term measure of brain injury, exhibiting high sensitivity in detecting regions of the brain most susceptible to hypoxic ischemic insults such as the basal ganglia, thalamus, and watershed regions [10]. Additionally, the brain MRI serves as a very good predictor for long‐term neurodevelopmental disabilities [9, 11].

For temperature correction, we used an algorithm to re‐analyze the reported pCO_2_ measurements taking the core body temperature of the newborn into account when calculating the revised pCO_2_ measurement. This type of pCO_2_ measurement corresponds to the pH‐stat method, whereas the reported pCO_2_ not corrected for body temperature obtained from the automated blood gas analyzers corresponds to the alpha‐stat method. The formulas the GEM automated blood gas analyzers employs are as follows [12].

pHT=pH=t−37×−0.01470.0065+×7.4−pH/0.019×T−37PCO2T=PCO2×10/×T−37PO2 T=PO2×10

where T = temperature entered by the operator and K = temporary subordinate calculation.

### 2.1. Statistical Analysis

Descriptive data were expressed using means and standard deviations (SDs) if normally distributed or median and interquartile range (IQR) if not normally distributed. A paired *t*‐test was performed to assess the significance of the mean difference in the lowest pCO_2_ among all patients in our cohort, comparing the alpha‐stat and pH‐stat blood gas analyses. Subsequently, a Bhapkar test was carried out to assess differences in the classification of patients as hypocapnic, normocapnic, or hypercapnic between the two methods based on their unique pCO_2_ readings.

Patients were stratified based on HIE severity. Sensitivity, specificity, positive predictive value (PPV), and negative predictive value (NPV) were calculated for the two methods of analysis. A multivariable logistic regression was performed, controlled for sex and gestational age. All statistical analysis were conducted using STATA 11th edition software. The study was approved by the Conjoint Health Research Ethics Board of the University of Calgary.

## 3. Results

We included 112 neonates diagnosed with HIE who received 72 h of TH. The mean gestational age (GA) was 39 weeks (SD 1.7). Among them, 15 (13%) were diagnosed with moderate HIE and the remaining 97 (87%) with severe HIE. Within the first week of life, 28 neonates (25%) had MRI evidence of moderate brain injury, while 84 (75%) displayed severe brain injury. The baseline characteristics and short‐term outcomes at discharge are described in Table 1.

**Table 1:** Baseline characteristics of study population.

**Baseline characteristics,** **N** = 112	
Mean gestational age (weeks) (SD)	39.0 (1.7)
Male, *n* (%)	57 (51)
Moderate HIE, *n* (%)	15 (13)
Severe HIE, *n* (%)	97 (87)
Median Apgar score at 5 min, *n* (IQR)	4.0 (4)
Median time of initiation of cooling (h) (IQR)	5.0 (2.8)
Median base excess within first hour of birth (mmol/L) (IQR)	10.1(9.2)
Median cord pH within first hour of birth (IQR)	7.0 (0.3)
Moderate brain injury, *n* (%)	84 (75)
Severe brain injury, *n* (%)	28 (25)
Mortality, *n* (%)	9 (8)
Composite outcome of severe brain injury on days 3–5 of life/death, *n* (%)	30 (27)

Abbreviations: IQR, interquartile range; SD, standard deviation.

PCO_2_ readings measured by the alpha‐stat and pH‐stat methods in neonates with moderate and severe HIE and their correlations are shown in Table 2. It is evident that mean difference in PCO_2_ measurements at different stages of HIE was significantly different between alpha‐stat and pH‐stat readings.

**Table 2:** Association of the alpha‐stat and pH‐stat methods on the mean hypocapnic pCO_2_ reading during therapeutic hypothermia.

**Clinical stage of HIE**	**Mean alpha-stat reading of PaCO**_**2**_ **in mmHg (SD)**	**Mean pH-stat reading of PaCO**_**2**_ **in mmHg (SD)**	**Mean difference in mmHg (95% CI)**	*p* **value**
All (*n* = 112)	30.1 ± 6.0	25.8 ± 6.7	4.3 (4.0‐4.6)	< 0.0001
Moderate HIE (*n* = 15)	28.9 ± 8.1	25.3 ± 7.5	3.6 (3.1‐4.1)	< 0.0001
Severe HIE (*n* = 97)	30.2 ± 6.2	25.8 ± 6.8	4.4 (3.0‐5.8)	< 0.0001

Abbreviations: CI, confidence interval; SD, standard deviation; TH, therapeutic hypothermia.

**Figure 1:**
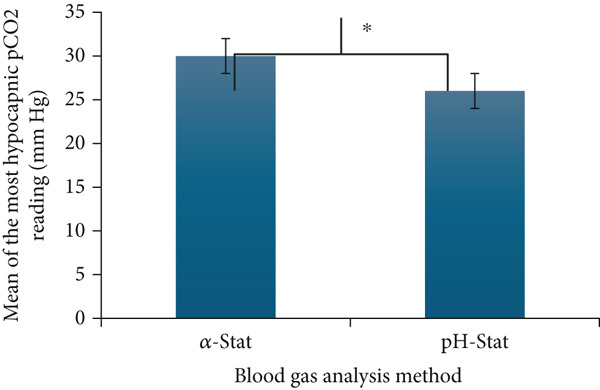
Comparing the mean of the most hypocapnic (lowest) pCO_2_ measurements for all the patients (*N* = 112) using the alpha‐stat and pH‐stat approach.

The mean and standard deviation of the single most hypocapnic pCO_2_ during TH for all patients in our cohort was 30.1 mmHg (6.2 mmHg) using the alpha‐stat approach but 25.8 mmHg (6.8 mmHg) using the pH‐stat approach. The mean difference in lowest pCO_2_ corresponding to the alpha‐stat and pH‐stat method was 4.3 mmHg (95% CI 4.0–4.6; *p* < 0.0001) (Figure 1). The median and interquartile range of temperature at the most hypocapnic pCO_2_ reading was 33.5°C (0.5). Further, in all the subgroups, the corresponding mean pCO_2_ was significantly lower with the pH‐stat approach compared to the alpha‐stat approach (Table 3).

**Table 3:** Classification of patients according to most hypocapnic PCO_2_ readings based on alpha‐stat and pH‐stat blood gas interpretation approaches.

**Alpha-stat**	**pH-stat**		
	**Hypocapnia (** **n** = 104 **)**	**Normocapnia (** **n** = 7**)**	**Hypercapnia (** **n** = 1**)**
Hypocapnia (PaCO_2_ < 35 mmHg)	82	0	0
Normocapnia (35 mmHg < PaCO_2_ < 45 mmHg)	22	4	0
Hypercapnia (PaCO_2_ > 45 mmHg)	0	3	1

*Note:* Bhapkar test, test for marginal homogeneity: overall *p* value < 0.0001.

Considering PaCO_2_, 25 patients (23%) were reclassified with the use of the pH‐stat approach. In 22 out of these 25 cases, the alpha‐stat approach classified the patients as “normocapnic” but the pH‐stat method classified them as “hypocapnic” (Table 4). The proportion of patients classified as hypocapnic, normocapnic, or hypercapnic was statistically different between the two approaches, with a significant downward reclassification associated with the pH‐stat approach (*p* < 0.0001) (Table 4).

**Table 4:** Classification of moderate and severe HIE according to the MRI outcomes.

**Method**		**Moderate HIE according to the MRI outcomes**			**Severe HIE according to the MRI outcomes**		
		**Severe brain injury**	**Non-severe brain injury**	**Total**	**Severe brain injury**	**Non-severe brain injury**	**Total**
Alpha‐stat	Hypocapnia	1 (TP)	10 (FP)	11	24 (TP)	46 (FP)	70
	No hypocapnia	0 (FN)	4 (TN)	4	3 (FN)	24 (TN)	27
	Total	1	14	15	27	70	97

pH‐stat	Hypocapnia	1 (TP)	13 (FP)	14	26 (TP)	64 (FP)	90
	No hypocapnia	0 (FN)	1 (TN)	1	1 (FN)	6 (TN)	7
	Total	1	14	15	27	70	97

|Abbreviations: FN, false negative; FP, false positive; TN, true negative; TP, true positive.

**Figure 2:**
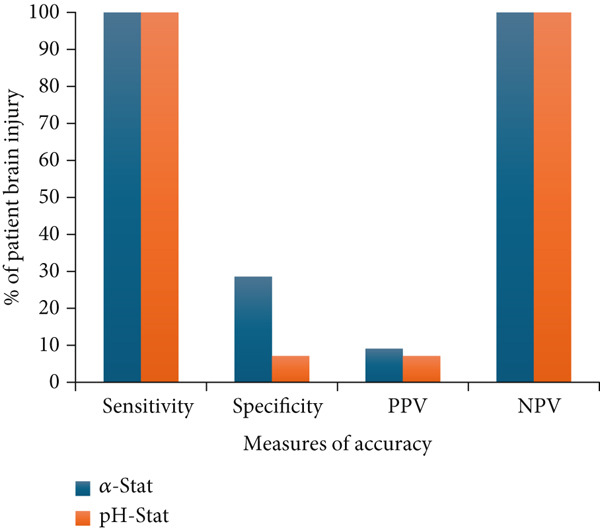
The percent of patient brain injury status (moderate or severe brain injury) correctly identified based on various measures of accuracy for moderate HIE patients (*N* = 15).

Among patients with moderate HIE (*n* = 15), the sensitivity and specificity of the two approaches for predicting the severity of brain injury based on classification of patients into the “hypocapnia” or “normocapnia/hypercapnia” group were not significantly different (Figure 2). The positive predictive values of both approaches were not significantly different (*p* = 0.35). We were unable to compare the two negative predictive values associated with the two approaches given the small number of patients classified as normocapnic/hypercapnic: four for alpha‐stat and one for pH‐stat (Table 4).

Among patients with severe HIE (*n* = 97), the sensitivity and specificity of the alpha‐stat approach for correctly identifying extent of brain injury was 88.9% (95% CI 70.8–97.6) and 34.3% (95% CI 23.3–46.2), respectively. In contrast, the sensitivity and specificity of the pH‐stat approach was 96.29% (95% CI 81.03–99.91) and 8.57% (95% CI 3.21–17.73), respectively. The positive predictive values were significantly higher with the alpha‐stat approach compared to the pH‐stat approach (*p* = 0.015). Negative predictive values did not differ significantly (*p* > 0.05).

After controlling for sex and gestational age, patients classified as hypocapnic by the alpha‐stat approach had an odds ratio of 4.40 (95% CI 1.19–16.27) for severe brain injury as compared to those classified as normocapnic/hypercapnic. Neonates classified as hypocapnic by the pH‐stat approach had an odds ratio of 2.94 (95% CI 1.15–26.48) for severe brain injury compared to those classified as normocapnic/hypercapnic.

## 4. Discussion

This was a retrospective cohort study assessing the ability of the alpha‐stat and pH‐stat blood gas analysis methods to predict the severity of brain injury. In both adjusted and unadjusted models, there were no significant differences between the alpha‐stat and pH‐stat method to predict severity of brain injury in neonates with HIE who underwent TH.

The findings are not unexpected given majority of the patients in our cohort had severe HIE (87%) to begin with. According to a systematic review by Ergenekon et al., TH significantly reduces the extent of brain injury in patients with moderate HIE but is less beneficial in patients with severe HIE [13]. It is believed that if the asphyxia insult is severe enough, as is often the case in severe HIE, the subsequent extent of brain injury is largely dependent on the severity and duration of the initial asphyxia insult itself, and the impact of TH on patient outcome is limited [13]. Therefore, since TH does not play a significant role in determining patient outcome for those with severe HIE, it may explain why no significant differences were observed in the ability of the two blood gas methods to predict severity of brain injury. Furthermore, according to the study by Pappas et al., although a single low pCO_2_ was associated with adverse outcomes in patients with HIE, the risk of developing severe neurologic disability increased with greater cumulative pCO_2_ < 35 mmHg exposure [14]. These findings suggest that using a cumulative exposure to pCO_2_ < 35 mmHg may be more informative in evaluating differences between the two blood gas analysis methods rather than a single time point of pCO_2_ instead. Most clinical studies comparing the alpha‐stat and pH‐stat method were performed in patients undergoing deep hypothermia with circulatory arrest, where patients were cooled to 18 °C leading to far more dramatic differences in pCO_2_ measurements between the two blood gas analysis methods compared to TH, where patients are cooled to 33–34 °C [8]. In these studies, the pH‐stat method was associated with better clinical outcomes than the alpha‐stat method [8]. Till date, three studies have been performed comparing the alpha‐stat vs. pH‐stat method for adult patients receiving TH following circulatory arrest, with two studies slightly favoring the alpha‐stat approach and one study showing no significance between the two methods in terms of patient outcome [15]. However, these findings may not be applicable to our study because the mode of cerebral injury vastly differs in newborns and adults.

The pH‐stat method was associated with a significant downward re‐classification of patients from “normocapnic” to “hypocapnic.” From a theoretical perspective, this was to be expected because in TH, the temperature of neonates is cooled to 33–34 °C and the pH‐stat method uses an algorithm to re‐analyze the crude ABG analyzer measurement for pH, pCO_2_, and pO_2_ by considering the true core body temperature versus the alpha‐stat method, which assumes a core body temperature of 37 °C. Cooling reduces the partial pressure of CO_2_ by increasing the solubility of gas in the blood. Thus, the alpha‐stat method, which uses the crude measurements from the ABG analyzer will always overestimate the pCO_2_. The appropriate classification of patients is critical to subsequent clinical decisions and patient management. Hypocapnia (pCO_2_ ≤ 35 mmHg) is associated with adverse neurological outcomes in neonates with HIE. Therefore, the significant downward re‐classification associated with the pH‐stat method is an important finding because it demonstrates that we classify and potentially manage these patients differently using the pH‐stat blood gas analysis method versus the alpha‐stat method.

Our study has several strengths. We obtained a complete dataset for all the patients in our cohort, thus eliminating the need to adjust or account for any missing values. Further, a trained neuroradiologist, who was blinded to the details of the study, evaluated the severity of the brain injury. Also, the study had good internal validity because all the procedures of TH were performed at either Alberta Children’s Hospital or Foothills Medical Centre. This ensured that a set protocol was used in the management of the newborns with HIE. However, there are several limitations that need to be taken into consideration as well. For example, the small sample size restricted the number of explanatory variables that could be adjusted for despite having identified several confounding variables that could affect the severity of brain injury. Further, although MRI is the most effective test for detecting the severity of brain injury, like any test, it is not a 100% accurate and could lead to false positive or false negatives when determining if injury is moderate or severe. We also could not establish inter‐rater validity as only one neuroradiologist examined the MRI scans. This limitation could be easily addressed by using multiple neuroradiologists to examine the MRI scans. Another limitation of this study is that, because our analysis focused on the lowest pCO_2_ value, we did not have a sufficiently complete dataset of serial pCO_2_ measurements to calculate the duration or number of hypocapnic events. Therefore, only the lowest pCO_2_ was used in this study. Finally, given that this was not a multi‐geographic study, further investigations are needed to confirm or refute the findings of our study.

In conclusion, our study found that there was no significant difference between the pH‐stat method and the alpha‐stat blood gas analysis method to predict severity of brain injury in neonates undergoing TH. However, the pH‐stat method was associated with a significant downward re‐classification of patients based on their pCO_2_ compared to the alpha‐stat method. In the future, comparing the ability of the two blood gas analysis methods to predict long‐term neurodevelopmental outcomes will provide further insight into the clinical outcomes of these patients with HIE undergoing TH. Given that TH yields the most benefit for patients with moderate HIE, perhaps evaluating the role of the two blood gas analysis methods exclusively in neonates with HIE may shed more light on the differences between the two methods.

## Conflicts of Interest

The authors declare no conflicts of interest.

## Author Contributions

V.G., Y.R., and A.K.L. conceptualized and wrote the study protocol, conducted the study, performed data analysis and interpretation, and drafted the manuscript. A.L. reviewed the manuscript and assisted in data collection. K.M., A.K.L., and Y.R. provided expert opinions and reviewed the manuscript. V.G., A.K., K.M., A.K.L., and Y.R. collectively finalized the manuscript.

## Funding

No funding was received for this manuscript.

## Data Availability

In accordance with the Alberta Health Services Data Disclosure Agreement, we are not able to provide or make the data available for any purpose to a third party without the prior written consent of Alberta Health Services, Calgary, Alberta.
